# Supplementary material for: Low immunogenicity of malaria pre‐erythrocytic stages can be overcome by vaccination
Source: EMBO Mol Med. 2021 Mar 11;13(4):e13390. doi: 10.15252/emmm.202013390 (PMC8033512; doi:10.15252/emmm.202013390)
Supplement: Supplementary file 1 — Appendix [file EMMM-13-e13390-s001.pdf]

Appendix

Table of content:

Appendix Figure S1: Flow cytometry gating strategies for identifying Kb-SIINFEKL+ effector CD8+ T cells from blood, spleen and liver.

Appendix Figure S2: Flow cytometry gating strategies for identifying IFN-γ+, TNF+, IL-2+ CD8+ T cells from the spleen and liver.

Appendix Figure S3: No peptide restimulation controls for splenic and liver CD8+ T cells from immunised mice, and peripheral blood CD8+ T cells from vaccinated mice.

Appendix Table S1: Numbers of animals used for Fig 2C

Appendix Table S2: Numbers of animals used for Fig 3C-D

Appendix Table S3: Numbers of animals used for Fig 3F-G and Fig EV4

Appendix Table S4: Exact levels of all p-values

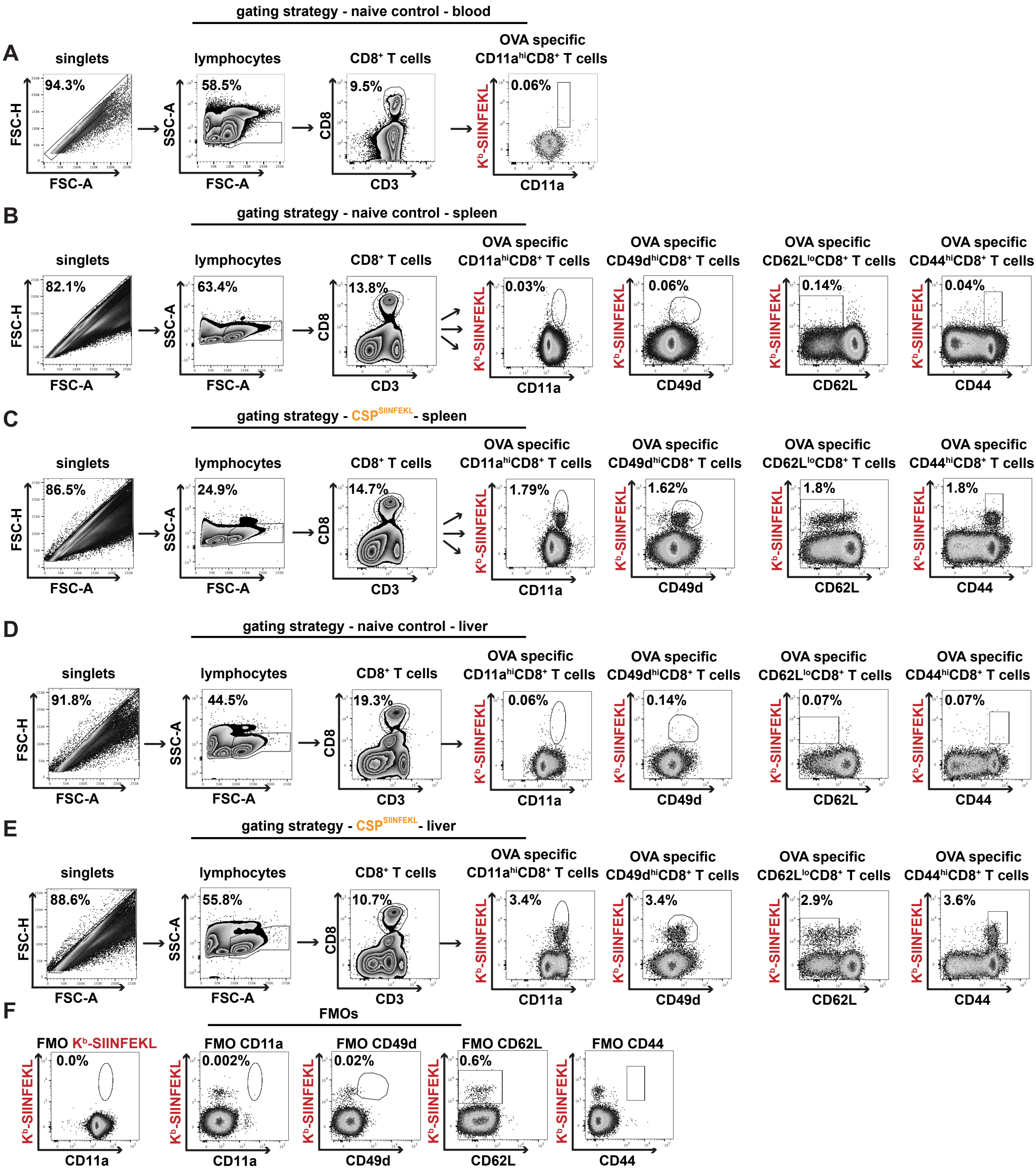

Appendix Figure S1: Flow cytometry gating strategies for identifying K^{b}-SIINFEKL+ effector CD8+ T cells from blood, spleen and liver.

(A) Example of flow cytometry gating panel for blood from naïve control mice. **(B,C)** Flow cytometry gating strategy showing examples of splenic CD8+ T cells from **(B)** naïve mice and **(C)** mice immunised with CSP^{SIINFEKL} parasites and OT-I cells. **(D,E)** Flow cytometry gating strategy showing examples of CD8+ T cells form the livers of **(D)** naïve mice and **(E)** mice immunised with CSP^{SIINFEKL} parasites and OT-I cells. **(F)** Example fluorescence minus one (FMO) controls from the blood of immunised mice used to gate cell populations for markers of effector phenotype (CD11a^{hi}, CD49d^{hi}, CD62L^{lo}, CD44^{hi}).

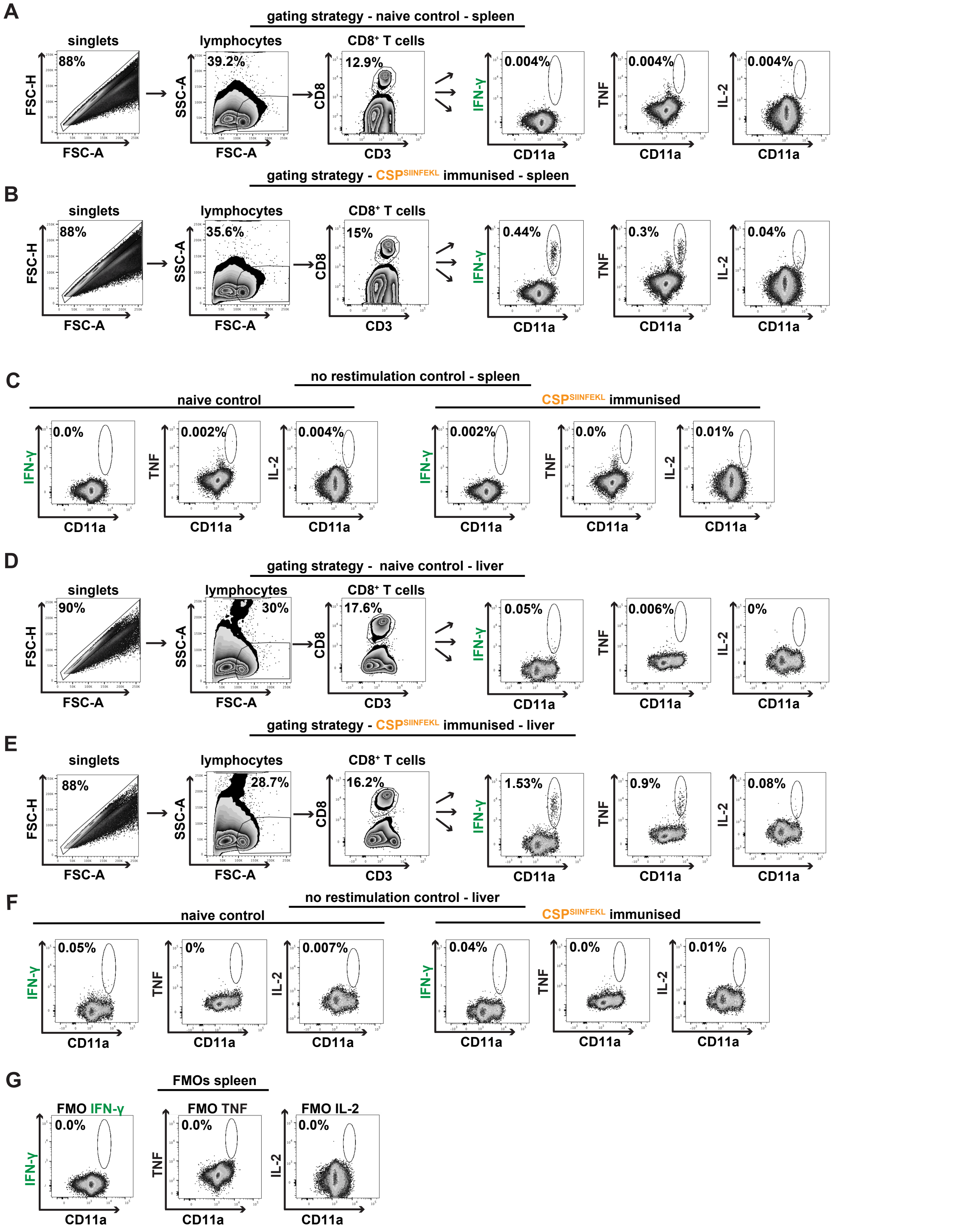

Appendix Figure S2: Flow cytometry gating strategies for identifying IFN-γ+, TNF+, IL-2+ CD8+ T cells from the spleen and liver.

(A,B) Flow cytometry gating strategy showing examples of splenic CD8+ T cells from **(A)** naïve mice and **(B)** mice immunised with CSPSIINFEKL parasites restimulated with SIINFEKL peptide. **(C)** Flow cytometry gating strategy showing example responses of splenic CD8+ T cells from naïve mice (left) and mice immunised with CSPSIINFEKL parasites (right) without peptide restimulation. **(D,E)** Flow cytometry gating strategy showing examples of CD8+ T cells from the livers of **(D)** naïve mice and **(E)** mice immunised with CSPSIINFEKL parasites and restimulated with SIINFEKL peptide. **(F)** Flow cytometry gating strategy showing example responses of CD8+ T cells from the livers of naïve mice (left) and mice immunised with CSPSIINFEKL parasites (right) without peptide restimulation. **(G)** Example fluorescence minus one (FMO) controls from the spleen of immunised mice

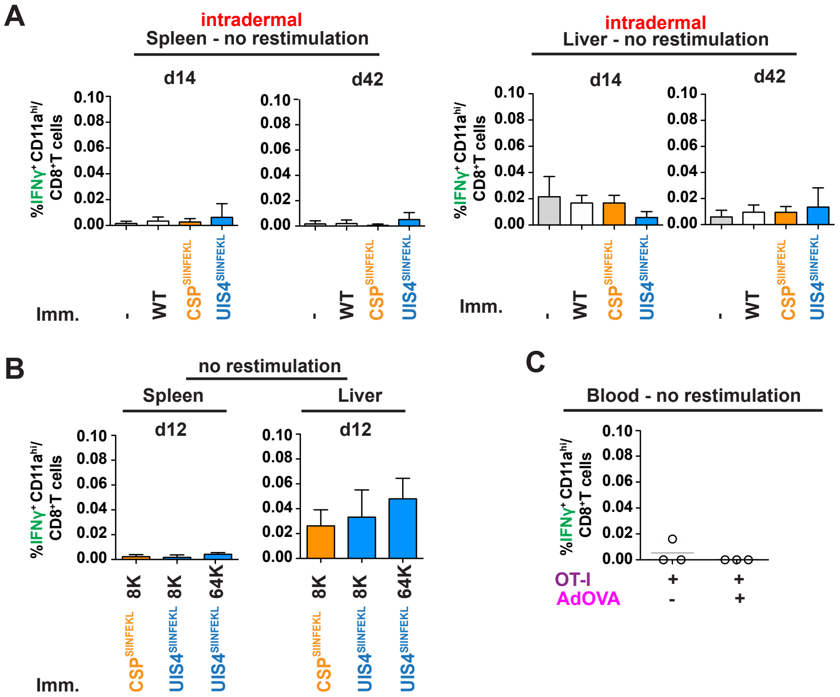

Appendix Figure S3: No peptide restimulation controls for splenic and liver CD8+ T cells from immunised mice, and peripheral blood CD8+ T cells from vaccinated mice.
(A) Bar charts showing the percentage of IFN-γ+ CD11a^{hi} CD8+ T cells following no peptide restimulation of lymphocytes from the spleen (left) and liver (right) at 14 and 42 days after intra-dermal immunisation of C57BL/6 mice with 10,000 CSP^{SIINFEKL} or UIS4^{SIINFEKL} sporozoites. **(B)** Bar charts showing the percentage of IFN-γ+ CD11a^{hi} CD8+ T cells following no peptide restimulation of lymphocytes from the spleen and liver at 12 days after immunisation of C57BL/6 mice with 8,000 CSP^{SIINFEKL} or UIS4^{SIINFEKL} sporozoites or 64,000 sporozoites UIS4^{SIINFEKL}.
(C) Scatter plot showing the percentage of IFN-γ+ CD11a^{hi} CD8+ T cells following no peptide restimulation of lymphocytes from the blood of mice vaccinated with 1x10^{8} ifu recombinant AdHu5 expressing whole ovalbumin (AdOVA) and/or 2x10^{6} OT-I splenocytes.

Appendix Table S1: Number of animals used for Fig 2C

	naïve controls	WT	CSP^{SIINFEKL}	UIS4^{SIINFEKL}
day 4	5	9	8	10
day 7	4	6	8	8
day 14	4	6	8	8
day 21	2	3	4	4
day 42	2	3	4	4
day 88	4	3	7	10

Appendix Table S2: Number of animals used for Fig 3C-D

	OT-I	WT + OT-I	CSP^{SIINFEKL} + OT-I	UIS4^{SIINFEKL} + OT-I
liver d14	2	3	4	4
spleen d14	2	3	4	4
liver d42	2	3	4	4
spleen 42	2	3	4	4

Appendix Table S3: Number of animals used for Fig 3F-G and Fig EV4

	OT-I	WT + OT-I	CSP^{SIINFEKL} + OT-I	UIS4^{SIINFEKL} + OT-I
liver d14	4	6	4	4
spleen d14	4	6	4	4
liver d42	4	6	4	4
spleen 42	4	6	4	4

Appendix Table S4: Exact levels of all p-values

Figure	Groups		Significant?	Symbol	p-Value
Figure 2C					
	Day 4	$CSP^{SIINFEKL}$ vs. $UIS4^{SIINFEKL}$	Yes	***	0.000506
	Day 7	$CSP^{SIINFEKL}$ vs. $UIS4^{SIINFEKL}$	Yes	**	0.003834
	Day 14	$CSP^{SIINFEKL}$ vs. $UIS4^{SIINFEKL}$	Yes	*	0.023608
	Day 21	$CSP^{SIINFEKL}$ vs. $UIS4^{SIINFEKL}$	No	ns	0.202371
	Day 42	$CSP^{SIINFEKL}$ vs. $UIS4^{SIINFEKL}$	Yes	*	0.044158
	Day 88	$CSP^{SIINFEKL}$ vs. $UIS4^{SIINFEKL}$	Yes	*	0.020426
Figure 3C					
	Liver day 14 (%)	OT-1 vs. WT + OT-1	No	ns	>0.9999
		OT-1 vs. $CSP^{SIINFEKL}$ + OT-1	No	ns	0.0673
		OT-1 vs. $UIS4^{SIINFEKL}$ + OT-1	No	ns	0.99
		WT + OT-1 vs. $CSP^{SIINFEKL}$ + OT-1	Yes	*	0.0415
		WT + OT-1 vs. $UIS4^{SIINFEKL}$ + OT-1	No	ns	0.9926
		$CSP^{SIINFEKL}$ + OT-1 vs. $UIS4^{SIINFEKL}$ + OT-1	Yes	*	0.0435
	Liver day 42 (%)	OT-1 vs. WT + OT-1	No	ns	0.9243
		OT-1 vs. $CSP^{SIINFEKL}$ + OT-1	No	ns	0.3563
		OT-1 vs. $UIS4^{SIINFEKL}$ + OT-1	No	ns	0.9609
		WT + OT-1 vs. $CSP^{SIINFEKL}$ + OT-1	No	ns	0.0918
		WT + OT-1 vs. $UIS4^{SIINFEKL}$ + OT-1	No	ns	0.9974
		$CSP^{SIINFEKL}$ + OT-1 vs. $UIS4^{SIINFEKL}$ + OT-1	No	ns	0.0899
	Spleen day 14 (%)	OT-1 vs. WT + OT-1	No	ns	0.9997
		OT-1 vs. $CSP^{SIINFEKL}$ + OT-1	No	ns	0.0538
		OT-1 vs. $UIS4^{SIINFEKL}$ + OT-1	No	ns	0.9894
		WT + OT-1 vs. $CSP^{SIINFEKL}$ + OT-1	Yes	*	0.0369
		WT + OT-1 vs. $UIS4^{SIINFEKL}$ + OT-1	No	ns	0.9949
		$CSP^{SIINFEKL}$ + OT-1 vs. $UIS4^{SIINFEKL}$ + OT-1	Yes	*	0.0369
	Spleen day 42 (%)	OT-1 vs. WT + OT-1	No	ns	0.9995
		OT-1 vs. $CSP^{SIINFEKL}$ + OT-1	No	ns	0.136
		OT-1 vs. $UIS4^{SIINFEKL}$ + OT-1	No	ns	0.9824
		WT + OT-1 vs. $CSP^{SIINFEKL}$ + OT-1	No	ns	0.1014
		WT + OT-1 vs. $UIS4^{SIINFEKL}$ + OT-1	No	ns	0.9914
		$CSP^{SIINFEKL}$ + OT-1 vs. $UIS4^{SIINFEKL}$ + OT-1	No	ns	0.1167
Figure 3D					
	Liver day 14 (absol. #)	OT-1 vs. WT + OT-1	No	ns	0.9982
		OT-1 vs. $CSP^{SIINFEKL}$ + OT-1	Yes	*	0.013
		OT-1 vs. $UIS4^{SIINFEKL}$ + OT-1	No	ns	0.91
		WT + OT-1 vs. $CSP^{SIINFEKL}$ + OT-1	Yes	**	0.008
		WT + OT-1 vs. $UIS4^{SIINFEKL}$ + OT-1	No	ns	0.9456
		$CSP^{SIINFEKL}$ + OT-1 vs. $UIS4^{SIINFEKL}$ + OT-1	Yes	*	0.0113
	Liver day 42 (absol. #)	OT-1 vs. WT + OT-1	No	ns	0.9938
		OT-1 vs. $CSP^{SIINFEKL}$ + OT-1	No	ns	0.7689
		OT-1 vs. $UIS4^{SIINFEKL}$ + OT-1	No	ns	0.9983
		WT + OT-1 vs. $CSP^{SIINFEKL}$ + OT-1	No	ns	0.5267
		WT + OT-1 vs. $UIS4^{SIINFEKL}$ + OT-1	No	ns	0.9994

		$CSP^{SIINFEKL}$ + OT-1 vs. $UIS4^{SIINFEKL}$ + OT-1	No	ns	0.5346
	Spleen day 14 (absol. #)	OT-1 vs. WT + OT-1	No	ns	0.9987
		OT-1 vs. $CSP^{SIINFEKL}$ + OT-1	Yes	*	0.0409
		OT-1 vs. $UIS4^{SIINFEKL}$ + OT-1	No	ns	0.9766
		WT + OT-1 vs. $CSP^{SIINFEKL}$ + OT-1	Yes	*	0.0297
		WT + OT-1 vs. $UIS4^{SIINFEKL}$ + OT-1	No	ns	0.9919
		$CSP^{SIINFEKL}$ + OT-1 vs. $UIS4^{SIINFEKL}$ + OT-1	Yes	*	0.031
	Spleen day 42 (absol. #)	OT-1 vs. WT + OT-1	No	ns	0.9997
		OT-1 vs. $CSP^{SIINFEKL}$ + OT-1	No	ns	0.1482
		OT-1 vs. $UIS4^{SIINFEKL}$ + OT-1	No	ns	0.9943
		WT + OT-1 vs. $CSP^{SIINFEKL}$ + OT-1	No	ns	0.1088
		WT + OT-1 vs. $UIS4^{SIINFEKL}$ + OT-1	No	ns	0.9981
		$CSP^{SIINFEKL}$ + OT-1 vs. $UIS4^{SIINFEKL}$ + OT-1	No	ns	0.1051
Figure 3F					
	Liver day 14 (%)	OT-1 vs. WT + OT-1	No	ns	>0.9999
		OT-1 vs. $CSP^{SIINFEKL}$ + OT-1	Yes	**	0.0029
		OT-1 vs. $UIS4^{SIINFEKL}$ + OT-1	No	ns	0.9775
		WT + OT-1 vs. $CSP^{SIINFEKL}$ + OT-1	Yes	**	0.0013
		WT + OT-1 vs. $UIS4^{SIINFEKL}$ + OT-1	No	ns	0.9693
		$CSP^{SIINFEKL}$ + OT-1 vs. $UIS4^{SIINFEKL}$ + OT-1	Yes	**	0.0061
	Liver day 42 (%)	OT-1 vs. WT + OT-1	No	ns	>0.9999
		OT-1 vs. $CSP^{SIINFEKL}$ + OT-1	No	ns	0.0697
		OT-1 vs. $UIS4^{SIINFEKL}$ + OT-1	No	ns	>0.9999
		WT + OT-1 vs. $CSP^{SIINFEKL}$ + OT-1	Yes	*	0.0429
		WT + OT-1 vs. $UIS4^{SIINFEKL}$ + OT-1	No	ns	>0.9999
		$CSP^{SIINFEKL}$ + OT-1 vs. $UIS4^{SIINFEKL}$ + OT-1	No	ns	0.0719
	Spleen day 14 (%)	OT-1 vs. WT + OT-1	No	ns	>0.9999
		OT-1 vs. $CSP^{SIINFEKL}$ + OT-1	Yes	**	0.0085
		OT-1 vs. $UIS4^{SIINFEKL}$ + OT-1	No	ns	0.9932
		WT + OT-1 vs. $CSP^{SIINFEKL}$ + OT-1	Yes	**	0.0051
		WT + OT-1 vs. $UIS4^{SIINFEKL}$ + OT-1	No	ns	0.9935
		$CSP^{SIINFEKL}$ + OT-1 vs. $UIS4^{SIINFEKL}$ + OT-1	Yes	*	0.0133
	Spleen day 42 (%)	OT-1 vs. WT + OT-1	No	ns	>0.9999
		OT-1 vs. $CSP^{SIINFEKL}$ + OT-1	Yes	*	0.0341
		OT-1 vs. $UIS4^{SIINFEKL}$ + OT-1	No	ns	0.9985
		WT + OT-1 vs. $CSP^{SIINFEKL}$ + OT-1	Yes	*	0.0208
		WT + OT-1 vs. $UIS4^{SIINFEKL}$ + OT-1	No	ns	0.999
		$CSP^{SIINFEKL}$ + OT-1 vs. $UIS4^{SIINFEKL}$ + OT-1	Yes	*	0.0457
Figure 3G					
	Liver day 14 (absol. #)	OT-1 vs. WT + OT-1	No	ns	>0.9999
		OT-1 vs. $CSP^{SIINFEKL}$ + OT-1	Yes	**	0.0012
		OT-1 vs. $UIS4^{SIINFEKL}$ + OT-1	No	ns	0.971
		WT + OT-1 vs. $CSP^{SIINFEKL}$ + OT-1	Yes	***	0.0005
		WT + OT-1 vs. $UIS4^{SIINFEKL}$ + OT-1	No	ns	0.9704
		$CSP^{SIINFEKL}$ + OT-1 vs. $UIS4^{SIINFEKL}$ + OT-1	Yes	**	0.0027
	Liver day 42 (absol. #)	OT-1 vs. WT + OT-1	No	ns	>0.9999
		OT-1 vs. $CSP^{SIINFEKL}$ + OT-1	No	ns	0.2334
		OT-1 vs. $UIS4^{SIINFEKL}$ + OT-1	No	ns	>0.9999

		WT + OT-1 vs. CSP^{SIINFEKL} + OT-1	No	ns	0.1793
		WT + OT-1 vs. UIS4^{SIINFEKL} + OT-1	No	ns	>0.9999
		CSP^{SIINFEKL} + OT-1 vs. UIS4^{SIINFEKL} + OT-1	No	ns	0.2429
	Spleen day 14 (absol. #)	OT-1 vs. WT + OT-1	No	ns	>0.9999
		OT-1 vs. CSP^{SIINFEKL} + OT-1	Yes	*	0.0228
		OT-1 vs. UIS4^{SIINFEKL} + OT-1	No	ns	0.9958
		WT + OT-1 vs. CSP^{SIINFEKL} + OT-1	Yes	*	0.0142
		WT + OT-1 vs. UIS4^{SIINFEKL} + OT-1	No	ns	0.9953
		CSP^{SIINFEKL} + OT-1 vs. UIS4^{SIINFEKL} + OT-1	Yes	*	0.0332
	Spleen day 42 (absol. #)	OT-1 vs. WT + OT-1	No	ns	>0.9999
		OT-1 vs. CSP^{SIINFEKL} + OT-1	Yes	*	0.0145
		OT-1 vs. UIS4^{SIINFEKL} + OT-1	No	ns	0.9983
		WT + OT-1 vs. CSP^{SIINFEKL} + OT-1	Yes	**	0.0079
		WT + OT-1 vs. UIS4^{SIINFEKL} + OT-1	No	ns	0.9984
		CSP^{SIINFEKL} + OT-1 vs. UIS4^{SIINFEKL} + OT-1	Yes	*	0.02
	Liver day 14 (%)	WT vs. CSP^{SIINFEKL}	Yes	***	0.0007
		WT vs. UIS4^{SIINFEKL}	No	ns	>0.9999
		WT vs. naive	No	ns	0.9949
		CSP^{SIINFEKL} vs. UIS4^{SIINFEKL}	Yes	***	0.0006
		CSP^{SIINFEKL} vs. naive	Yes	***	0.0004
		UIS4^{SIINFEKL} vs. naive	No	ns	0.9962
	Liver day 42 (%)	WT vs. CSP^{SIINFEKL}	Yes	***	<0.0001
		WT vs. UIS4^{SIINFEKL}	No	ns	0.8561
		WT vs. naive	No	ns	0.9995
		CSP^{SIINFEKL} vs. UIS4^{SIINFEKL}	Yes	***	<0.0001
		CSP^{SIINFEKL} vs. naive	Yes	***	<0.0001
		UIS4^{SIINFEKL} vs. naive	No	ns	0.9028
	Spleen day 14 (%)	WT vs. CSP^{SIINFEKL}	Yes	***	0.0002
		WT vs. UIS4^{SIINFEKL}	No	ns	0.9973
		WT vs. naive	No	ns	0.9998
		CSP^{SIINFEKL} vs. UIS4^{SIINFEKL}	Yes	***	0.0003
		CSP^{SIINFEKL} vs. naive	Yes	***	0.0002
		UIS4^{SIINFEKL} vs. naive	No	ns	0.9994
	Spleen day 42 (%)	WT vs. CSP^{SIINFEKL}	Yes	***	<0.0001
		WT vs. UIS4^{SIINFEKL}	No	ns	0.7535
		WT vs. naive	No	ns	0.6135
		CSP^{SIINFEKL} vs. UIS4^{SIINFEKL}	Yes	***	<0.0001
		CSP^{SIINFEKL} vs. naive	Yes	***	<0.0001
		UIS4^{SIINFEKL} vs. naive	No	ns	0.1412
Figure 4D					
	Liver day 14 (absol. #)	WT vs. CSP^{SIINFEKL}	Yes	***	<0.0001
		WT vs. UIS4^{SIINFEKL}	No	ns	0.9982
		WT vs. naive	No	ns	0.9975
		CSP^{SIINFEKL} vs. UIS4^{SIINFEKL}	Yes	***	<0.0001
		CSP^{SIINFEKL} vs. naive	Yes	***	<0.0001
		UIS4^{SIINFEKL} vs. naive	No	ns	0.9837
	Liver day 42 (absol. #)	WT vs. CSP^{SIINFEKL}	Yes	***	<0.0001

		WT vs. UIS4^{SIINFEKL}	No	ns	0.9826
		WT vs. naive	No	ns	>0.9999
		CSP^{SIINFEKL} vs. UIS4^{SIINFEKL}	Yes	***	<0.0001
		CSP^{SIINFEKL} vs. naive	Yes	***	<0.0001
		UIS4^{SIINFEKL} vs. naive	No	ns	0.9884
	Spleen day 14 (absol. #)	WT vs. CSP^{SIINFEKL}	Yes	***	<0.0001
		WT vs. UIS4^{SIINFEKL}	No	ns	0.9901
		WT vs. naive	No	ns	>0.9999
		CSP^{SIINFEKL} vs. UIS4^{SIINFEKL}	Yes	***	<0.0001
		CSP^{SIINFEKL} vs. naive	Yes	***	<0.0001
		UIS4^{SIINFEKL} vs. naive	No	ns	0.9895
	Spleen day 42 (absol. #)	WT vs. CSP^{SIINFEKL}	Yes	***	<0.0001
		WT vs. UIS4^{SIINFEKL}	No	ns	0.8939
		WT vs. naive	No	ns	0.6887
		CSP^{SIINFEKL} vs. UIS4^{SIINFEKL}	Yes	***	<0.0001
		CSP^{SIINFEKL} vs. naive	Yes	***	<0.0001
		UIS4^{SIINFEKL} vs. naive	No	ns	0.289
Figure 4F					
	Liver day 14 (%)	naive vs. WT	No	ns	>0.9999
		naive vs. CSP^{SIINFEKL}	Yes	*	0.0109
		naive vs. UIS4^{SIINFEKL}	No	ns	0.9995
		WT vs. CSP^{SIINFEKL}	Yes	**	0.0099
		WT vs. UIS4^{SIINFEKL}	No	ns	0.9983
		CSP^{SIINFEKL} vs. UIS4^{SIINFEKL}	Yes	*	0.0132
	Liver day 42 (%)	naive vs. WT	No	ns	>0.9999
		naive vs. CSP^{SIINFEKL}	No	ns	0.08
		naive vs. UIS4^{SIINFEKL}	No	ns	0.999
		WT vs. CSP^{SIINFEKL}	No	ns	0.0801
		WT vs. UIS4^{SIINFEKL}	No	ns	0.999
		CSP^{SIINFEKL} vs. UIS4^{SIINFEKL}	No	ns	0.1009
	Spleen day 14 (%)	naive vs. WT	No	ns	>0.9999
		naive vs. CSP^{SIINFEKL}	Yes	*	0.018
		naive vs. UIS4^{SIINFEKL}	No	ns	0.9962
		WT vs. CSP^{SIINFEKL}	Yes	*	0.0184
		WT vs. UIS4^{SIINFEKL}	No	ns	0.9967
		CSP^{SIINFEKL} vs. UIS4^{SIINFEKL}	Yes	*	0.0264
	Spleen day 42 (%)	naive vs. WT	No	ns	>0.9999
		naive vs. CSP^{SIINFEKL}	Yes	*	0.0131
		naive vs. UIS4^{SIINFEKL}	No	ns	0.9993
		WT vs. CSP^{SIINFEKL}	Yes	*	0.0118
		WT vs. UIS4^{SIINFEKL}	No	ns	0.9976
		CSP^{SIINFEKL} vs. UIS4^{SIINFEKL}	Yes	*	0.0163
Figure 4G					
	Liver day 14 (absol. #)	naive vs. WT	No	ns	>0.9999
		naive vs. CSP^{SIINFEKL}	Yes	*	0.0412
		naive vs. UIS4^{SIINFEKL}	No	ns	0.9983
		WT vs. CSP^{SIINFEKL}	Yes	*	0.0394
		WT vs. UIS4^{SIINFEKL}	No	ns	0.9974

		$CSP^{SIINFEKL}$ vs. $UIS4^{SIINFEKL}$	No	ns	0.0549
	Liver day 42 (absol. #)	naive vs. WT	No	ns	>0.9999
		naive vs. $CSP^{SIINFEKL}$	Yes	*	0.0271
		naive vs. $UIS4^{SIINFEKL}$	No	ns	0.9906
		WT vs. $CSP^{SIINFEKL}$	Yes	*	0.0272
		WT vs. $UIS4^{SIINFEKL}$	No	ns	0.9908
		$CSP^{SIINFEKL}$ vs. $UIS4^{SIINFEKL}$	Yes	*	0.0453
	Spleen day 14 (absol. #)	naive vs. WT	No	ns	>0.9999
		naive vs. $CSP^{SIINFEKL}$	Yes	*	0.0326
		naive vs. $UIS4^{SIINFEKL}$	No	ns	0.9989
		WT vs. $CSP^{SIINFEKL}$	Yes	*	0.0329
		WT vs. $UIS4^{SIINFEKL}$	No	ns	0.999
		$CSP^{SIINFEKL}$ vs. $UIS4^{SIINFEKL}$	Yes	*	0.0417
	Spleen day 42 (absol. #)	naive vs. WT	No	ns	0.9999
		naive vs. $CSP^{SIINFEKL}$	Yes	*	0.0173
		naive vs. $UIS4^{SIINFEKL}$	No	ns	>0.9999
		WT vs. $CSP^{SIINFEKL}$	Yes	*	0.0154
		WT vs. $UIS4^{SIINFEKL}$	No	ns	0.9997
		$CSP^{SIINFEKL}$ vs. $UIS4^{SIINFEKL}$	Yes	*	0.018
Figure 5C					
	Liver day 12 (%)	$CSP^{SIINFEKL}$ 8K vs. $UIS4^{SIINFEKL}$ 8K	Yes	***	<0.0001
		$CSP^{SIINFEKL}$ 8K vs. $UIS4^{SIINFEKL}$ 64K	Yes	***	<0.0001
		$UIS4^{SIINFEKL}$ 8K vs. $UIS4^{SIINFEKL}$ 64K	No	ns	0.9639
	Spleen day 12 (%)	$CSP^{SIINFEKL}$ 8K vs. $UIS4^{SIINFEKL}$ 8K	Yes	***	<0.0001
		$CSP^{SIINFEKL}$ 8K vs. $UIS4^{SIINFEKL}$ 64K	Yes	***	<0.0001
		$UIS4^{SIINFEKL}$ 8K vs. $UIS4^{SIINFEKL}$ 64K	No	ns	0.9982
	Liver day 12 (absol. #)	$CSP^{SIINFEKL}$ 8K vs. $UIS4^{SIINFEKL}$ 8K	Yes	***	<0.0001
		$CSP^{SIINFEKL}$ 8K vs. $UIS4^{SIINFEKL}$ 64K	Yes	***	<0.0001
		$UIS4^{SIINFEKL}$ 8K vs. $UIS4^{SIINFEKL}$ 64K	No	ns	0.8979
	Spleen day 12 (absol. #)	$CSP^{SIINFEKL}$ 8K vs. $UIS4^{SIINFEKL}$ 8K	Yes	***	<0.0001
		$CSP^{SIINFEKL}$ 8K vs. $UIS4^{SIINFEKL}$ 64K	Yes	***	<0.0001
		$UIS4^{SIINFEKL}$ 8K vs. $UIS4^{SIINFEKL}$ 64K	No	ns	0.9561
Figure 6D					
		WT vacc v WT nonvacc	No	ns	0.1142
		$CSP^{SIINFEKL}$ vacc v $CSP^{SIINFEKL}$ nonvacc	Yes	**	0.0095
		$UIS4^{SIINFEKL}$ vacc v $UIS4^{SIINFEKL}$ nonvacc	Yes	**	0.0055
Figure EV1C					
		WT vs. $CSP^{SIINFEKL}$	No	ns	0.2643
		WT vs. $UIS4^{SIINFEKL}$	No	ns	0.705
		$CSP^{SIINFEKL}$ vs. $UIS4^{SIINFEKL}$	No	ns	0.715
Figure EV1D					
		WT vs. $CSP^{SIINFEKL}$	No	ns	0.2737
		WT vs. $UIS4^{SIINFEKL}$	No	ns	0.9867
		$CSP^{SIINFEKL}$ vs. $UIS4^{SIINFEKL}$	No	ns	0.3514
Figure EV1E					
	24 hours	WT vs. $CSP^{SIINFEKL}$	No	ns	0.0771
		WT vs. $UIS4^{SIINFEKL}$	No	ns	0.434
		$CSP^{SIINFEKL}$ vs. $UIS4^{SIINFEKL}$	No	ns	0.5243

	48 hours	WT vs. CSP^{SIINFEKL}	No	ns	0.1794
		WT vs. UIS4^{SIINFEKL}	No	ns	0.3633
		CSP^{SIINFEKL} vs. UIS4^{SIINFEKL}	No	ns	0.8655
	72 hours	WT vs. CSP^{SIINFEKL}	Yes	*	0.0314
		WT vs. UIS4^{SIINFEKL}	No	ns	0.0791
		CSP^{SIINFEKL} vs. UIS4^{SIINFEKL}	No	ns	0.972
	CD49d	naive vs. UIS4^{SIINFEKL}	No	ns	0.7181
		naive vs. CSP^{SIINFEKL}	Yes	***	<0.0001
		naive vs. WT	No	ns	>0.9999
		UIS4^{SIINFEKL} vs. CSP^{SIINFEKL}	Yes	***	<0.0001
		UIS4^{SIINFEKL} vs. WT	No	ns	0.752
		CSP^{SIINFEKL} vs. WT	Yes	***	<0.0001
	CD11a	naive vs. UIS4^{SIINFEKL}	No	ns	0.6539
		naive vs. CSP^{SIINFEKL}	Yes	***	<0.0001
		naive vs. WT	No	ns	0.9985
		UIS4^{SIINFEKL} vs. CSP^{SIINFEKL}	Yes	***	<0.0001
		UIS4^{SIINFEKL} vs. WT	No	ns	0.7452
		CSP^{SIINFEKL} vs. WT	Yes	***	<0.0001
	CD62L	naive vs. UIS4^{SIINFEKL}	No	ns	0.539
		naive vs. CSP^{SIINFEKL}	Yes	***	<0.0001
		naive vs. WT	No	ns	0.9866
		UIS4^{SIINFEKL} vs. CSP^{SIINFEKL}	Yes	***	<0.0001
		UIS4^{SIINFEKL} vs. WT	No	ns	0.7313
		CSP^{SIINFEKL} vs. WT	Yes	***	<0.0001
	CFSE	naive vs. UIS4^{SIINFEKL}	No	ns	0.4589
		naive vs. CSP^{SIINFEKL}	Yes	***	<0.0001
		naive vs. WT	No	ns	0.9961
		UIS4^{SIINFEKL} vs. CSP^{SIINFEKL}	Yes	***	<0.0001
		UIS4^{SIINFEKL} vs. WT	No	ns	0.5817
		CSP^{SIINFEKL} vs. WT	Yes	***	<0.0001
Figure EV4					
	TNF Liver day 14 (%)	OT-1 vs. WT + OT-1	No	ns	0.9965
		OT-1 vs. CSP^{SIINFEKL} + OT-1	Yes	**	0.0073
		OT-1 vs. UIS4^{SIINFEKL} + OT-1	No	ns	0.9148
		WT + OT-1 vs. CSP^{SIINFEKL} + OT-1	Yes	**	0.0054
		WT + OT-1 vs. UIS4^{SIINFEKL} + OT-1	No	ns	0.9582
		CSP^{SIINFEKL} + OT-1 vs. UIS4^{SIINFEKL} + OT-1	Yes	*	0.0249
	TNF Liver day 42 (%)	OT-1 vs. WT + OT-1	No	ns	0.9766
		OT-1 vs. CSP^{SIINFEKL} + OT-1	No	ns	0.0851
		OT-1 vs. UIS4^{SIINFEKL} + OT-1	No	ns	0.9991
		WT + OT-1 vs. CSP^{SIINFEKL} + OT-1	No	ns	0.1117
		WT + OT-1 vs. UIS4^{SIINFEKL} + OT-1	No	ns	0.9935
		CSP^{SIINFEKL} + OT-1 vs. UIS4^{SIINFEKL} + OT-1	No	ns	0.1072
	TNF Spleen day 14 (%)	OT-1 vs. WT + OT-1	No	ns	0.9975
		OT-1 vs. CSP^{SIINFEKL} + OT-1	Yes	*	0.0276
		OT-1 vs. UIS4^{SIINFEKL} + OT-1	No	ns	0.9616
		WT + OT-1 vs. CSP^{SIINFEKL} + OT-1	Yes	*	0.0235

		WT + OT-1 vs. UIS4^{SIINFEKL} + OT-1	No	ns	0.9857
		CSP^{SIINFEKL} + OT-1 vs. UIS4^{SIINFEKL} + OT-1	No	ns	0.0616
	TNF Spleen day 42 (%)	OT-1 vs. WT + OT-1	No	ns	0.9995
		OT-1 vs. CSP^{SIINFEKL} + OT-1	Yes	*	0.0269
		OT-1 vs. UIS4^{SIINFEKL} + OT-1	No	ns	0.9611
		WT + OT-1 vs. CSP^{SIINFEKL} + OT-1	Yes	*	0.0184
		WT + OT-1 vs. UIS4^{SIINFEKL} + OT-1	No	ns	0.9735
		CSP^{SIINFEKL} + OT-1 vs. UIS4^{SIINFEKL} + OT-1	No	ns	0.066
	IL-2 Liver day 14 (%)	OT-1 vs. WT + OT-1	No	ns	0.9994
		OT-1 vs. CSP^{SIINFEKL} + OT-1	Yes	**	0.0017
		OT-1 vs. UIS4^{SIINFEKL} + OT-1	No	ns	0.8692
		WT + OT-1 vs. CSP^{SIINFEKL} + OT-1	Yes	***	0.0009
		WT + OT-1 vs. UIS4^{SIINFEKL} + OT-1	No	ns	0.8862
		CSP^{SIINFEKL} + OT-1 vs. UIS4^{SIINFEKL} + OT-1	Yes	**	0.0071
	IL-2 Liver day 42 (%)	OT-1 vs. WT + OT-1	No	ns	0.8135
		OT-1 vs. CSP^{SIINFEKL} + OT-1	No	ns	0.5628
		OT-1 vs. UIS4^{SIINFEKL} + OT-1	No	ns	0.8948
		WT + OT-1 vs. CSP^{SIINFEKL} + OT-1	No	ns	0.1369
		WT + OT-1 vs. UIS4^{SIINFEKL} + OT-1	No	ns	0.9994
		CSP^{SIINFEKL} + OT-1 vs. UIS4^{SIINFEKL} + OT-1	No	ns	0.2248
	IL-2 Spleen day 14 (%)	OT-1 vs. WT + OT-1	No	ns	0.9841
		OT-1 vs. CSP^{SIINFEKL} + OT-1	Yes	**	0.0098
		OT-1 vs. UIS4^{SIINFEKL} + OT-1	No	ns	0.8803
		WT + OT-1 vs. CSP^{SIINFEKL} + OT-1	Yes	*	0.0101
		WT + OT-1 vs. UIS4^{SIINFEKL} + OT-1	No	ns	0.9677
		CSP^{SIINFEKL} + OT-1 vs. UIS4^{SIINFEKL} + OT-1	Yes	*	0.0341
	IL-2 Spleen day 42 (%)	OT-1 vs. WT + OT-1	No	ns	>0.9999
		OT-1 vs. CSP^{SIINFEKL} + OT-1	Yes	*	0.0104
		OT-1 vs. UIS4^{SIINFEKL} + OT-1	No	ns	0.9969
		WT + OT-1 vs. CSP^{SIINFEKL} + OT-1	Yes	**	0.0054
		WT + OT-1 vs. UIS4^{SIINFEKL} + OT-1	No	ns	0.9964
		CSP^{SIINFEKL} + OT-1 vs. UIS4^{SIINFEKL} + OT-1	Yes	*	0.0153
