# Supplementary material for: Low immunogenicity of malaria pre‐erythrocytic stages can be overcome by vaccination
Source: EMBO Mol Med. 2021 Mar 11;13(4):e13390. doi: 10.15252/emmm.202013390 (PMC8033512; doi:10.15252/emmm.202013390)
Supplement: Supplementary file 2 — Expanded View Figures PDF [file EMMM-13-e13390-s003.pdf]

Expanded View Figures

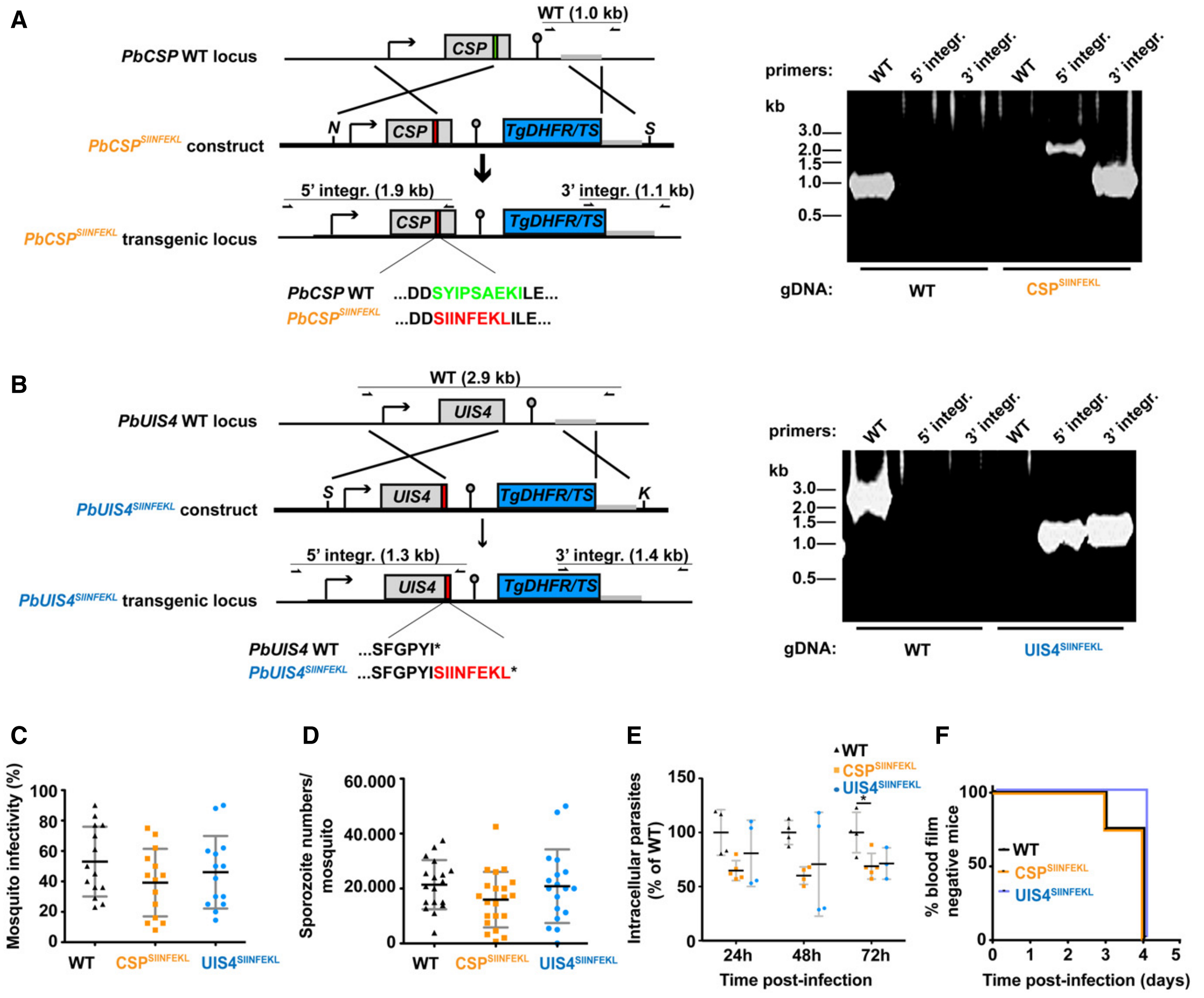

Figure EV1. Generation of transgenic CSP^{SIINFEKL} and UIS4^{SIINFEKL} *P. berghei* lines.

Plasmodium berghei parasites expressing the CD8^{+} T-cell epitope of ovalbumin, SIINFEKL, in the context of CSP or UIS4 were generated using double homologous recombination, combining drug-resistance selection (through incorporation of the *dhfr/ts* gene from *Toxoplasma gondii*) and cloning by limiting dilution to select for correctly recombined parasites.

A, B Diagrams illustrate the reverse genetics strategy. (A) In CSP^{SIINFEKL}, SIINFEKL replaces the immunodominant CD8^{+} T-cell epitope SYIPSAEK(I) of CSP. (B) In UIS4^{SIINFEKL} SIINFEKL is adjoined to the carboxyl-terminus of the UIS4 protein. Purified schizonts of WT *P. berghei* ANKA were transfected with linearised plasmid by electroporation as described (Janse *et al*, 2006), and immediately injected intravenously in the tail vein of a mouse. The day after transfection, pyrimethamine (70 mg/l) was orally administered in the drinking water for selection of transgenic parasites. Transgenic clones were generated in mice by *in vivo* cloning by limiting dilution. Correct integration of the constructs and purity of the transgenic lines were verified by diagnostic PCR using primer combinations specific for the unmodified *CSP* or *UIS4* locus, and for the 5' and 3' recombination events as indicated by lines, arrows and expected fragment sizes.

C Oocyst midgut infectivity of mosquitoes infected with WT, CSP^{SIINFEKL} or UIS4^{SIINFEKL}. The mean percentage (± SD) of infected midguts was enumerated 10–14 days after infection (14 biological replicates).

D Salivary glands were isolated from WT-, CSP^{SIINFEKL}- or UIS4^{SIINFEKL}-infected mosquitoes and mean sporozoite numbers (± SD) were enumerated between 18–30 days after infection (CSP^{SIINFEKL}, 21 biological replicates; UIS4^{SIINFEKL}, 18 biological replicates; WT, 18 biological replicates).

E Numbers of intracellular parasites of hepatoma cells 24, 48 and 72 h after infection with WT, CSP^{SIINFEKL} or UIS4^{SIINFEKL} sporozoites. The cells were fixed, stained and then were enumerated from two independent experiments. Values are percent of intracellular parasites compared with the mean of WT intracellular parasites ± SD (*$P < 0.05$; one-way ANOVA with Tukey's multiple comparison test). See Appendix Table S4 for exact *P*-values.

F Kaplan–Meier curve showing the prepatency of WT, CSP^{SIINFEKL} or UIS4^{SIINFEKL} sporozoite infections after intravenous injection of 800 sporozoites ($n = 4$ per group).

 © 2021 The Authors

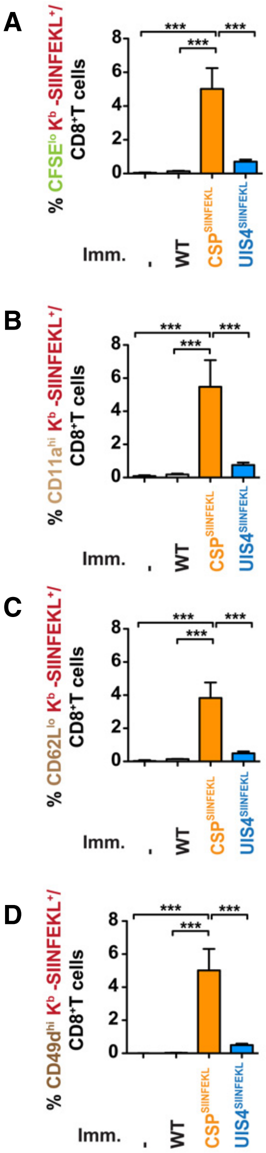

Figure EV2. Sporozoite surface antigen leads to greater expansion of antigen-specific CD8$^+$ T cells than EEF vacuolar membrane antigen.

A–D C57BL/6 mice (*n* = 4 per group), which received 2 × 10^6 CFSE-labelled OT-I splenocytes, were immunised with 10,000 γ-radiation attenuated WT, CSP^{SIINFEKL} or UIS4^{SIINFEKL} sporozoites intravenously. 5 days later, mice were sacrificed, spleens harvested and splenocytes assessed for (A) CFSE dilution of antigen-experienced K^b-SIINFEKL$^+$ CD8$^+$ T cells and stained *ex vivo* for effector CD8$^+$ T-cell surface markers (B) CD11a^{hi}, (C) CD62L^{lo} and (D) CD49d^{hi}. Bar charts show mean values (± SEM) from representative experiments (****P* < 0.001; one-way ANOVA with Tukey's multiple comparison test). See Appendix Table S4 for exact *P*-values.

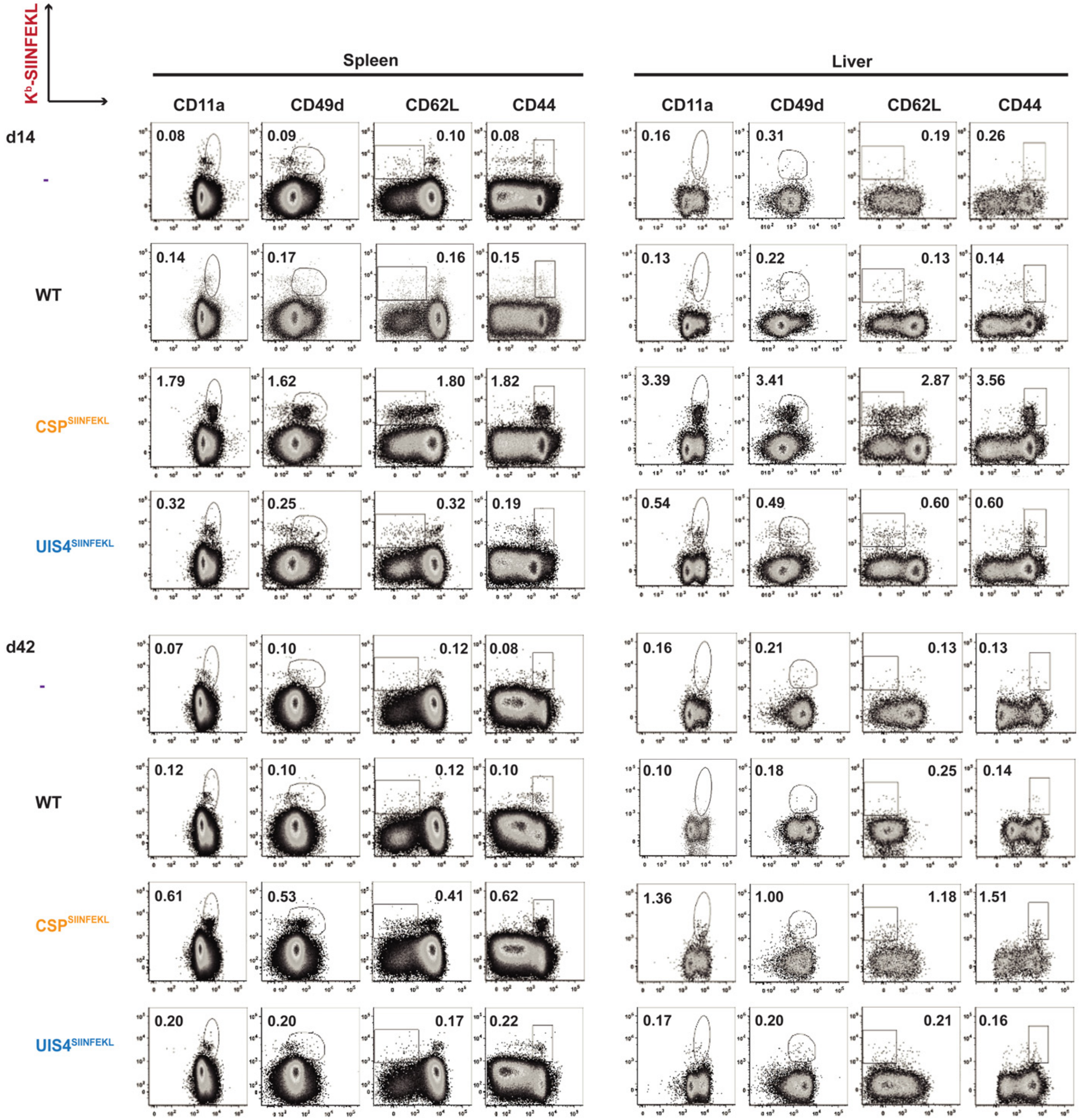

Figure EV3. Sporozoite surface antigen induces a greater effector CD8$^+$ T-cell phenotype than EEF vacuolar membrane antigen.

C57BL/6 mice received 2 × 10^6 OT-I cells alone (n = 2) or were additionally immunised with 10,000 γ-radiation attenuated WT (n = 3), CSP^{SIINFEKL} (n = 4) or UIS4^{SIINFEKL} (n = 4) sporozoites intravenously. Spleens and livers were harvested either 14 or 42 days later, and proportions of CD8$^+$ T cells expressing effector surface markers were quantified. Flow cytometry plots show representative percentages of CD8$^+$ T cells co-staining K^b-SIINFEKL and markers of effector phenotype (CD11a^{hi}, CD49d^{hi}, CD62L^{lo}, CD44^{hi}).

 © 2021 The Authors

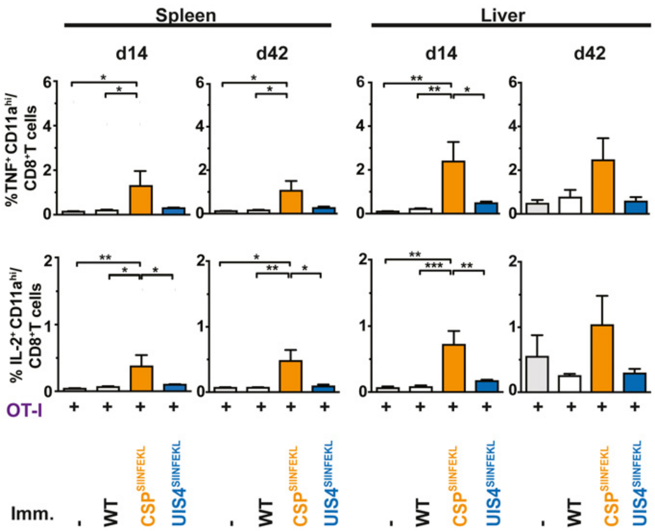

Figure EV4. Antigen-experienced SIINFEKL-specific CD8$^+$ T cells also produce TNF and IL-2.

C57BL/6 mice received 2 × 10^6 OT-I cells alone (n = 4) or were additionally immunised with 10,000 γ-radiation attenuated WT (n = 6), CSP^{SIINFEKL} (n = 4) or UIS4^{SIINFEKL} (n = 4) sporozoites intravenously. Spleens and livers were harvested either 14 or 42 days after immunisation and lymphocytes restimulated *ex vivo* with SIINFEKL peptide at 10 µg/ml per well for 5–6 h. The upper panel of bar charts show the percentage of CD11a^{hi} TNF secreting CD8$^+$ T cells, the bottom panel CD11a^{hi} IL-2 secreting CD8$^+$ T cells. This is a representation of one experiment from two experiments performed. Bar charts show mean values (±SEM) from representative experiments (*P < 0.05, **P < 0.01, ***P < 0.001; one-way ANOVA with Tukey's multiple comparison test). See Appendix Table S4 for exact P-values.

© 2021 The Authors
